# Oregano essential oil improves piglet health and performance through maternal feeding and is associated with changes in the gut microbiota

**DOI:** 10.1186/s42523-020-00064-2

**Published:** 2021-01-04

**Authors:** H. N. Hall, D. J. Wilkinson, M. Le Bon

**Affiliations:** 1 Anpario plc, Unit 5, Manton Wood, Worksop, S80 2RS UK; 2 Antimicrobial Resistance, Omics and Microbiota research group, Clifton Lane, Nottingham Trent University, Nottingham, NG11 8NS UK; 3 Nottingham Trent University, School of Animal, Rural and Environmental Sciences, Southwell, NG25 0QF UK

**Keywords:** Essential oil, Oregano, Pig, Microbiota, Microbial transfer, Performance, Creep feed, Oregano, Gut, Health

## Abstract

**Background:**

With a growing demand for safe and sustainable alternatives to antimicrobials, functional feed ingredients such as plant essential oils have been evaluated for their potential to improve gut health. Amongst these, oregano essential oil (OEO) with the main active compounds carvacrol and thymol has been reported to have antimicrobial and antioxidative properties resulting in improved intestinal barrier function and growth in pigs and poultry. However, its impact on the gut microbiota still remains unclear. The aim of this study was to examine the effect of an oregano essential oil phytobiotic on sow and piglet performance and faecal microbiota.

**Results:**

Piglets from OEO supplemented sows were significantly heavier at one week of age and showed a trend for improved average daily weight gain from birth to weaning. Post-weaning, maternally supplemented piglets were numerically heavier at 10 weeks post-weaning and at slaughter with a reduced variability in bodyweight. Health records showed that piglets in the OEO supplemented litters had significantly reduced incidence of therapeutic treatment and reduced mortality. In both sows and piglets, the structure and composition of the faecal microbiota varied considerably over time. Sows supplemented with OEO during lactation showed an increase in the relative abundance of *Lactobacillaceae* family. In addition, there was an increase in the relative abundance of families known to be important in fibre digestion (*Fibrobacteriaceae* and *Akkermansiaceae)*. Analysis of piglet microbiota at two weeks and four weeks of age revealed a relative decrease in *Enterobacteriaceae* while butyrate producers (*Lachnospiraceae* family) were increased at both timepoints.

**Conclusion:**

We hypothesise that the effects observed from this study were exerted through modulation of the gut microbial communities in the sow and her offspring through maternal microbial transfer. Understanding the link between the gut microbiota and dietary factors represents a keystone to improving health and performance for sustainable pig production. Reducing antimicrobial usage can help to reduce the risk of antimicrobial resistance (AMR) which is a global focus for animal production.

## Background

Pig production is under pressure to supply efficient protein, for an increasing human population, which is both high welfare and environmentally sustainable. There is a growing concern over antimicrobial resistance (AMR) [1] which has led to increasing demands for further reductions in prophylactic antimicrobial use. AMR is a natural evolutionary process but is known to be accelerated following misuse or overuse of antimicrobials [2]. This selective pressure can lead to the proliferation of a resistant bacterial population within the gut thereby increasing the potential for horizontal gene transfer between bacteria. Misuse of antimicrobials has been shown to increase the presence of antimicrobial resistance genes in the animal and human population such as MCR-1 [3, 4]. Natural solutions to improve animal health and reduce the reliance on antimicrobials are therefore increasingly important and essential oils could be one such solution.

In recent years, interest in the pig microbiota in relation to its composition, function and association with performance and health has been flourishing. Increased accessibility of high throughput technologies and improved affordability for large scale studies have enhanced our fundamental understanding on development and assembly of the pig microbiota over time and through production stages [5–10]. As with other species, the gut microbiota in pigs has been found to be strongly influenced by age and diet, which opens opportunities to target microbial communities for improved digestive health and efficiency using a non-antibiotic dietary approach.

Essential oils are the major group of phytogenic feed additives obtained from plants. These natural volatile compounds are isolated from plant material by methods such as steam distillation. They have been used in human and animal feeding for many years, historically for flavouring but more recently for their functional properties [11]. Oregano essential oil (OEO) specifically from *Origanum vulgare L.* is a relatively well understood phytogenic known to exhibit antimicrobial [12, 13], antioxidant [14] and anti-inflammatory properties [15]. OEO contains two main active components; carvacrol and thymol, both of which have been shown to be beneficial in improving pig gut health [16], modifying sow faecal bacteria populations and lactation performance [17–19].

This study aims to evaluate the effect of maternal OEO supplementation on sow and piglet microbiota during the pre-weaning period. With increasing evidence suggesting that colonisation of the bacterial community early in life can impact performance both in the short and long term [20], the effect of maternal OEO supplementation on animal health and performance was also evaluated from birth to slaughter. In addition, we investigated if exposure of OEO though maternal supplementation would increase piglet intake of creep also containing OEO.

## Results

Baseline data for all litters is provided in Additional File 1. Briefly, there were no significant differences in baseline parameters between groups allocated to control or OEO, except the number of sows requiring assistance during farrowing was lower in the OEO group (*p* < 0.05). However, farrowing was not monitored during night-time hours.

### Sow body condition and performance

Sow body condition scores (BCS) and the scoring index used are shown in Additional File 2. Scoring was adapted for commercial use [21]. At the start of the study, allocation to control or OEO group was balanced by BCS and parity of the sows, so that BCS was statistically similar between the groups at the pre-farrow timepoint (T0). At farrowing there was no effect of treatment but there was a positive effect of OEO on BCS at weaning (*p* = 0.034). Despite high temperatures experienced during the trial (Additional File 3), sow feed intake during lactation remained consistent (Table 1). There was no significant effect of OEO treatment on weekly feed intake, overall intake or on the number of refusals during lactation (Table 1). However, there was a trend for reduced intake before farrowing in the OEO group (*p* = 0.058). Sow performance post-weaning and subsequent parity fertility rates were conserved between treatments.

**Table 1:** Sow feed intake during lactation

	Control		Treatment (OEO)		*p* value
	Mean	SE	Mean	SE	
Sow feed intake pre-farrow (kg/d)	2.40^A^	0.03	2.31^B^	0.03	0.058
Sow feed intake week 1 (kg/d)	5.17	0.12	5.22	0.11	0.732
Sow feed intake week 2 (kg/d)	9.62	0.21	9.32	0.20	0.306
Sow feed intake week 3 (kg/d)	10.97	0.23	11.11	0.23	0.655
Sow feed intake week 4 (kg/d)	12.20	0.17	12.15	0.17	0.819
Sow feed average overall (kg/d)	7.75	0.08	7.82	0.12	0.647
Refusal number (meal)	11	–	8	–	0.649

*OEO* Oregano Essential Oil

*SE* Standard error

Superscript letters A-B represent statistical significance at *p* < 0.1

### Piglet growth performance

**Fig. 1:**
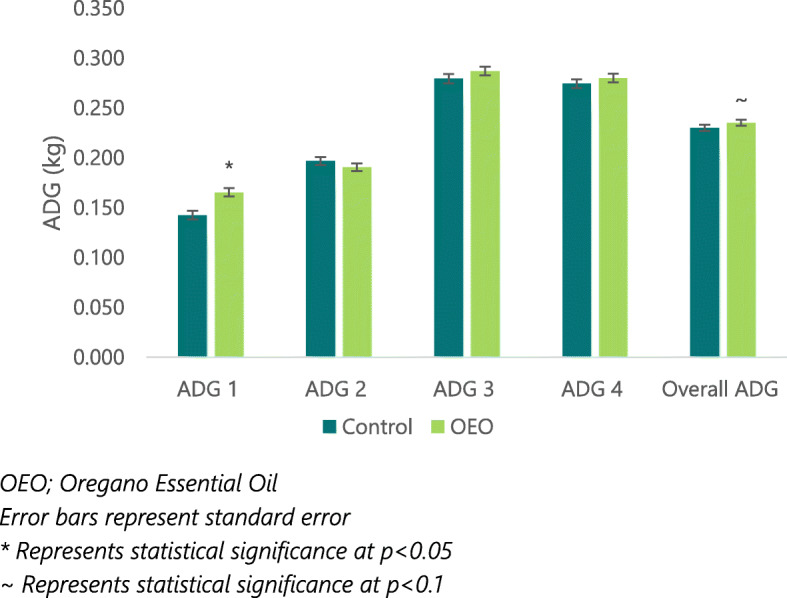
Piglet average daily gain (ADG) from birth to weaning

Average piglet weight and average daily gain (ADG) are shown in Fig. 1 and Table 2. Birth weight was not statistically affected by treatment, however, there was a significant increase in piglet body weight in the OEO supplement sow group at week 1 of age compared to the control (*p* = 0.006) resulting from a significant increase in ADG during the first week of life (*p* = 0.045). Repeated measures ANOVA showed a trend for increased ADG from birth to weaning in piglets from the OEO group (*p* = 0.079) (Fig. 1).

**Table 2:** Treatment effect on piglet growth performance

	Control		Treatment (OEO)		p value
	Mean	SE	Mean	SE	
Birth weight (kg)	1.36	0.02	1.38	0.02	0.374
Total litter birth weight (kg)	19.95	0.68	19.87	0.73	0.930
Weight week 1 (kg)	2.11^a^	0.03	2.23^b^	0.03	0.006
Weight week 2 (kg)	3.46	0.05	3.50	0.05	0.292
Weight week 3 (kg)	5.44	0.07	5.54	0.07	0.302
Weight week 4 (weaning) (kg)	7.37	0.09	7.55	0.09	0.156
Mean weight 10 weeks post weaning (kg)	40.76	0.38	41.32	0.40	0.396
ADG 10 weeks post weaning (kg/d)	0.476	0.005	0.483	0.005	0.305
Average slaughter age (day)	147.81	0.628	147.70	0.473	0.890
Mean slaughter weight (kg)	88.42	2.08	91.82	1.56	0.190

*OEO* Oregano Essential Oil

*ADG* Average daily gain

*SE* Standard error

Superscript letters a-b represent statistical significance at *p* < 0.05

There was no significant effect of treatment on weekly faecal score (Additional File 4). Results of faecal scoring indicated that there was no sign of scour during the trial. It is noted that an insufficient number of samples were found during the first week and that only partial coverage was recovered at week 2 and week 3, likely due to coprophagia.

Creep feed supplemented with OEO was offered to piglets from all sows from two weeks of age until 10 days post-weaning. Weekly creep feed intake more than doubled in week 3–4 compared to week 2–3. There was no significant increase in creep intake in the OEO group weekly or over the trial period (Table 3) and intakes were in line with other trials and commercial expectations [22, 23].

**Table 3:** Piglet creep feed intake

	Control		Treatment (OEO)		p value
	Mean	SE	Mean	SE	
Creep feed intake week 2–3 g/p/d	10.93	1.66	10.67	1.30	0.903
Creep feed intake week 3–4 g/p/d	22.15	3.09	23.59	2.27	0.706
Creep feed intake total week 2–4 g/p/d	231.53	30.42	239.83	20.54	0.820

*OEO* Oregano Essential Oil

*SE* Standard error

*g/pppd* gram per piglet per day

*g/pp* gram per piglet

**Fig. 2:**
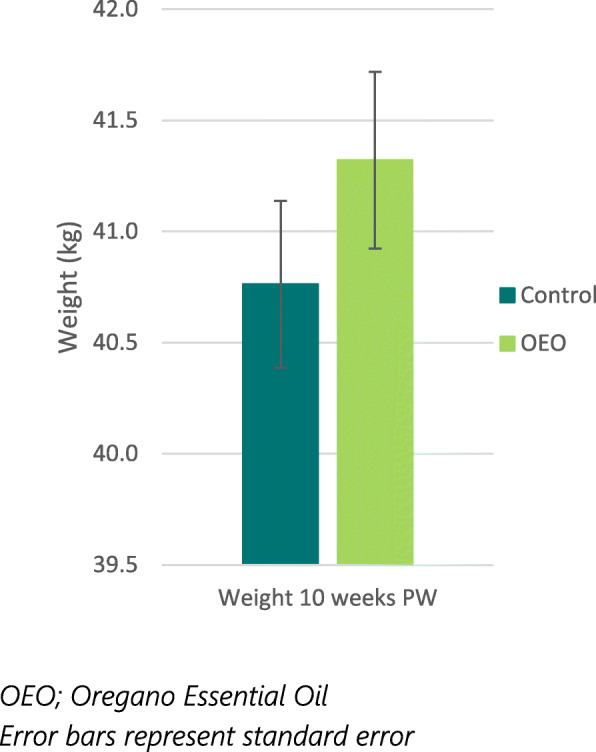
Average Pig weight 10 weeks post-weaning

Post-weaning, piglets were sorted according to size and mixed across treatments. On average piglets consumed 234 kg of creep feed per pen (60–72 pigs per pen) in the first 10 days post-weaning corresponding to an average of 329 g/d daily feed intake. At 10 weeks post-weaning (~ 14 weeks of age), pigs were individually weighed (Table 2, Fig. 2) and ADG post-weaning was calculated (Table 2). The majority (89%) of the pigs at this age still carried their ear tag for individual identification. There was no significant effect of maternal treatment on post-weaning performance at week 10 but piglets from OEO supplemented sows were numerically heavier.

**Fig. 3:**
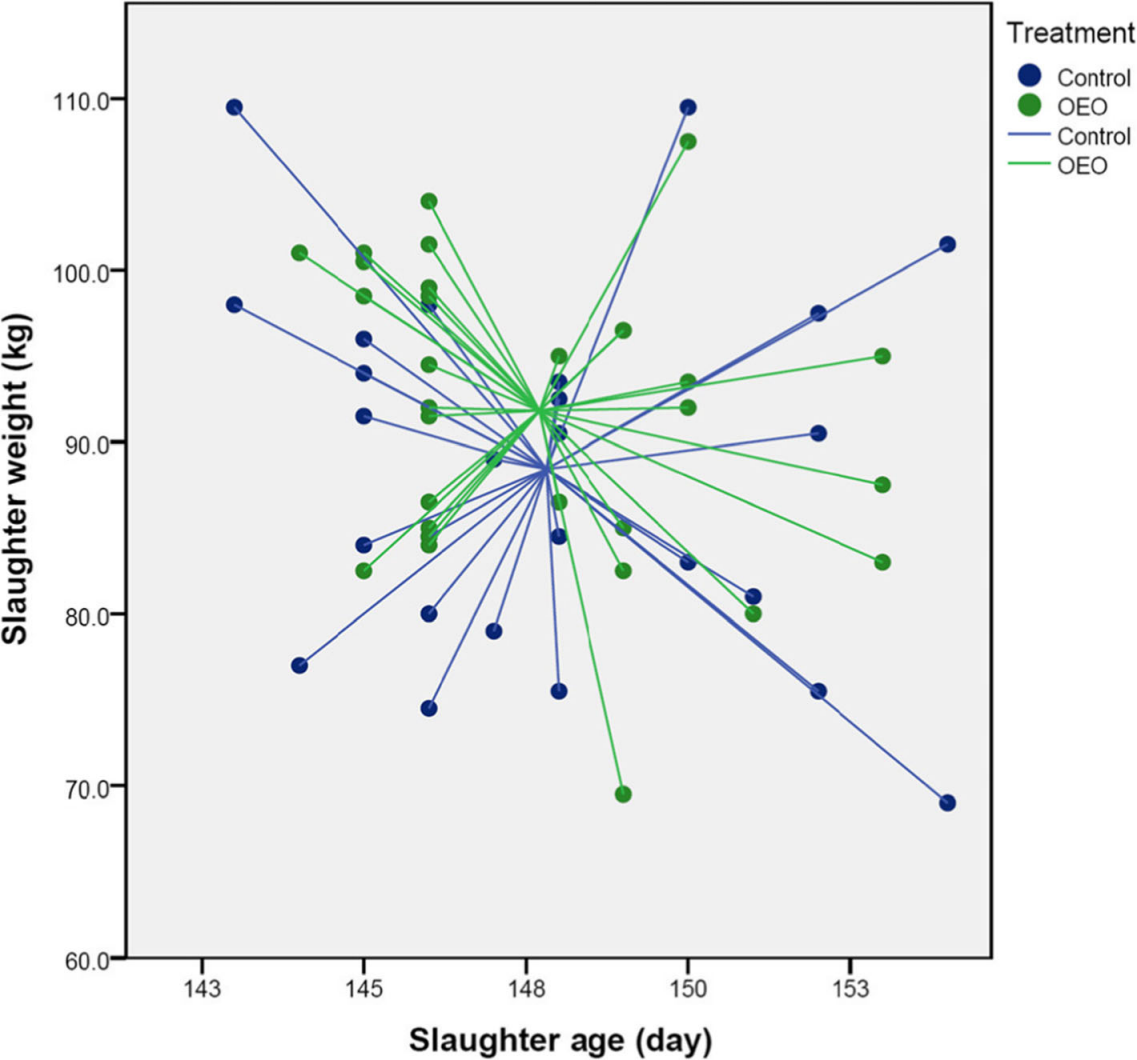
Slaughter weight against days to slaughter and centroid point for each group

A small number of pigs retained their ear tag until slaughter (*n* = 26, *n* = 30 for control and OEO groups, respectively) (Table 2). From this population, pigs from the OEO sow group finished on average 3.4 kg heavier for the same number of production days (~ 148 days), although this did not reach statistical significance (*p* > 0.05). Plot of weight against days to slaughter showed that the distribution of piglets from the OEO sow group was less disperse than the control pigs indicating a higher homogeneity across age and weight ranges (Fig. 3).

### Mortality and morbidity

Mortality was reduced in OEO supplemented litters by 3.4% from 14.3 to 10.9% (Table 4). Despite this numerical improvement, Chi-square statistics with Yates regression on counts showed that this did not reach statistical significance (*p* = 0.117). Suspected cause of death was recorded and showed that overall, the majority of piglet deaths were due to overlay (54.58%), followed by “unknown” causes (37.5%), and 8.18% were euthanised due to low viability or for welfare reasons. It was also noted that more than 75% of mortalities occurred before 5 days of age.

**Table 4:** Effect of treatment on morbidity and medication

	Control	Treatment (OEO)	*p* value
Total number of piglets born alive	441	428	–
Mortality: Total number of piglet death Pre-weaning	63	47	0.143
Pre-weaning mortality % (relative to total born alive)	14.3%	10.9%	–
Morbidity (total number of piglet)	51^A^	34^B^	0.072
Medication use (total number of piglet receiving intervention)	46	35	0.253
Medication use - weighted (total number of intervention)	64^a^	39^b^	0.003
Mortality: Total number of piglet death Post-weaning	7	9	0.632
Post weaning mortality % (relative to number of piglet weaned)	1.86%	2.36%	–

*OEO* Oregano Essential Oil

*Morbidity* Defined as recorded number of observation regarding piglet health

*Medication* Intervention was administered where necessary according to the farm standard welfare procedures

Superscript letters A-B represent statistical significance at p ≤ 0.1

Superscript letters a-b represent statistical significance at p ≤ 0.05

Health checks and medication use were recorded throughout the trial and were carried out blinded to the treatment group. Morbidity was defined as the recorded number of observations regarding individual piglet health such as lameness or swollen joints. Piglet morbidity tended to be reduced in the OEO sow group (*p* = 0.072). Not all observations required medical intervention but based on the duration and severity of the clinical signs, intervention was carried out according to the welfare principles and standard practices of the farm. Medication use was decreased in piglets from OEO supplemented sows resulting in a 4.2% reduction in therapeutic treatment prior to weaning (*p* = 0.003, Table 4). The main cause of medication was recorded as lameness or joint swelling of unknown cause and was treated via intramuscular injection to individual piglets.

### Bacterial community composition analysis by 16S rRNA gene sequencing in sows

**Fig. 4:**
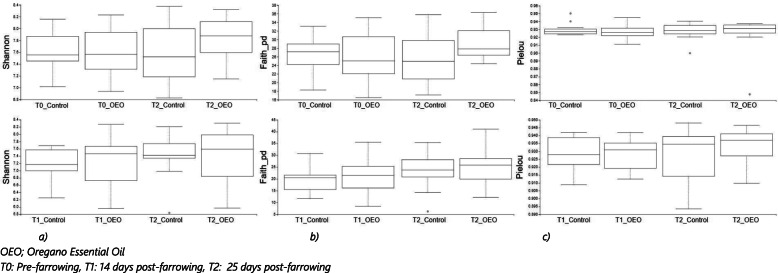
Faecal microbiota alpha diversity of sows (top panel) and piglets (bottom panel) across sampling time point and treatment represented by **a** Shannon, **b** Faith's Phylogenetic Diversity (Faith_PD) and c) Pileou’s Eveness (Pilou_E)

We evaluated the microbial community richness and diversity in sow and piglet faeces using Shannon index (a quantitative measure of community richness), Pielou’s Evenness (a measure of community evenness) and Faith’s Phylogenetic Diversity (a qualitative measure of community richness that incorporates phylogenetic relationships between the features) (Fig. 4). Before farrowing and at the start of OEO supplementation (T0), baseline diversity indices were similar between groups (*p* > 0.05). No significant differences were found in any of the alpha diversity indices between timepoints or treatment groups. However, it was noted that both Shannon and PD diversity values numerically decreased between T0 and 25 days post-farrowing (T2) in the control group while they increased in the OEO supplemented group.

**Fig. 5:**
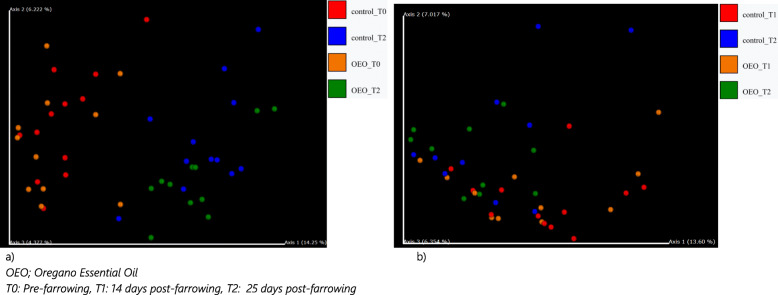
Principal coordinates analysis (PCoA) plots based on unweighted UniFrac distances by sampling time point and treatment for **a** sows and **b** piglets

The principal coordinate analysis (PCoA) based on the Bray–Curtis and unweighted UniFrac distance revealed that the samples clustered together according to timepoint, which indicated a shift in the gut bacterial community structure with the changes in the sow’s housing, nutrients and physiological state (Fig. 5, Additional File 5). PERMANOVA analysis confirmed significant separation of gut bacterial communities of sows between the timepoints (R-squared: 0.156 *p* < 0.001 for Bray Curtis, and R-squared: 0.138 *p* = 0.002 for Unweighted UniFrac). No significant effect of OEO treatment was shown in beta diversity of samples at T2 however, it was observed that samples from control sows were more widely distributed than samples from OEO supplemented sows at T2 (Fig. 5a).

**Fig. 6:**
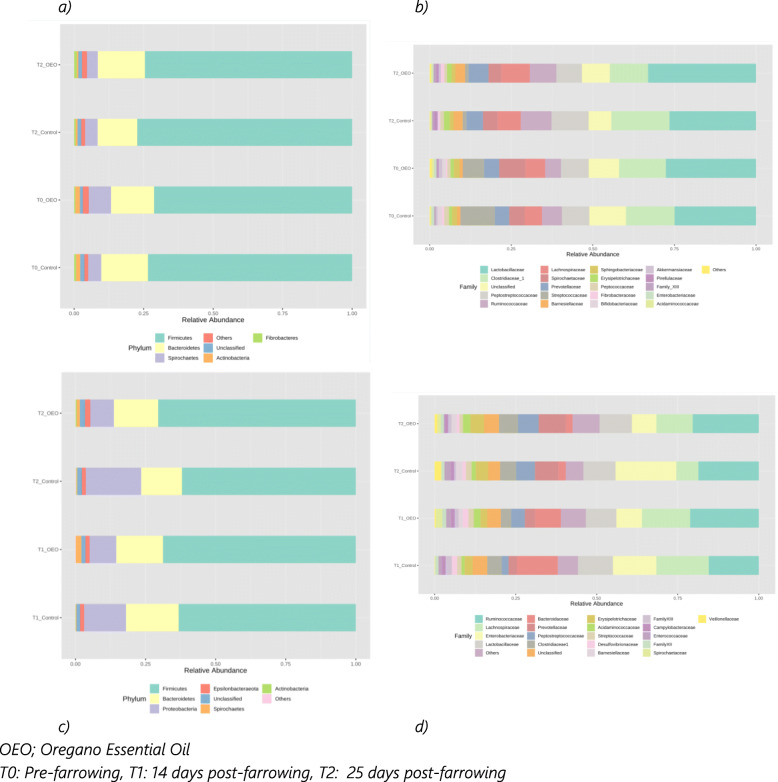
Relative abundance of microbial taxa for sows at the (**a**) phylum and (**b**) family level and piglets at the (**c**) phylum and (**d**) family level according to timepoints and treatment groups

Comparisons of the relative abundances of the microbiota compositions between sows from the control and OEO groups are shown in Fig. 6a-b. At the phylum level, a total of 18 phyla were observed in sow samples with *Firmicutes* (0.741 ± 0.047) being the most abundant phylum followed by *Bacteroidetes* (0.159 ± 0.028) and *Spirochaetes* (0.053 ± 0.029) across each stage and treatment (values in bracket indicates relative proportion mean ± 95% CI). At the family level, the top most abundant family in sow samples included *Lactobacillaceae* (0.280 ± 0.056), *Clostridiaceae*_1 (0.146 ± 0.041), *Peptostreptococcaceae* (0.088 ± 0.025), *Ruminococcaceae* (0.070 ± 0.032), *Lachnospiraceae* (0.068 ± 0.020), *Spirochaetaceae* (0.053 ± 0.029), *Prevotellaceae* (0.052 ± 0.009), *Streptococcaceae* (0.048 ± 0.072), *Barnesiellaceae* (0.017 ± 0.015) and *Erysipelotrichaceae* (0.014 ± 0.007).

In order to determine which microbial taxa contributed to the separation of the faecal microbiota between timepoints in sows (regardless of treatment), we performed linear discriminant analysis (LDA) effect size (LEfSe), which revealed that members of the *Bifidobacteriaceae*, *Spirochaetaceae* and *Streptococcaceae* family were more abundant at T0 than T2, while *Fibrobacteraceae, Erysipelotrichaeceae, Pirellulaceae, Barnesiellaceae and Ruminococcaceae* were more abundant at T2 compared to T0, at a threshold value of q < 0.1 (FDR) and LDA > 3.0 (Additional File 6).

**Fig. 7:**
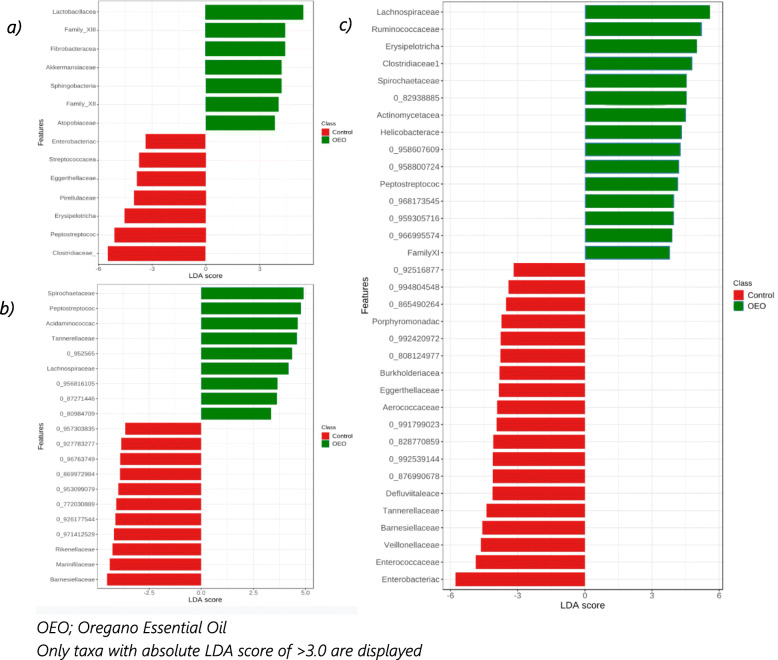
Linear discriminant analysis effect size (LEfSe) between control and OEO treatment at the family level for **a** Sows at 25 days post-farrowing (T2), **b** piglets at 14 days post-farrowing (T1), and **c** piglets at 25 days post-farrowing (T2)

Results of LEfSe at the family level revealed that the faeces of sows supplemented with OEO had higher abundance of *Lactobacillaceae, Clostridiales Family_XIII, Fibrobacteraceae, Akkermansiaceae, Sphingobacteriaceae, Clostridiales Family_XII and Atopobiaceae* and lower abundance of *Clostridiaceae, Peptostreptococcaceae, Erysipelotrichaceae, Pirellulaceae, Eggerthellaceae, Streptococcaceae* and *Enterobacteriaceae* than control sows at T2 (Fig. 7a).

### Bacterial community composition analysis by 16S rRNA gene sequencing in piglets

No significant differences in bacterial community richness and diversity were observed in any of the alpha diversity indices used between treatment groups and timepoint (*p* > 0.05), despite an overall numerical increase in piglet Shannon and PD diversity values between T1 and T2 (Fig. 4).

The principal coordinate analysis (PCoA) based on the Bray–Curtis and Unweighted UniFrac distance revealed that piglet faecal samples primarily clustered together according to timepoint, which indicated a shift in the bacterial community structure over time as a piglet matured and was offered creep feed supplementation (Fig. 5b, Additional File 5). PERMANOVA analysis confirmed significant separation of gut bacterial communities of piglets between the timepoints (R-squared: 0.103 *p* = 0.011 for Bray–Curtis, and R-squared: 0.045 *p* = 0.007 for Unweighted UniFrac). No significant effect of OEO treatment was shown in beta diversity of piglet’s faeces at 14 days (T1) or 25 days (T2) post-farrowing. As observed with sow samples, samples from control piglets were more disperse than those from piglets from the OEO supplemented litters at T1 and T2 (Fig. 5b, Additional File 5b).

Similar to sows, *Firmicutes* and *Bacteroidetes* were the most abundant phyla found in piglet samples however, in piglets this was followed by *Proteobacteria* and *Epsilonbacteraeota* (Fig. 6c). The relative abundance of *Proteobacteria* phylum in the piglet control group was markedly increased compared to samples from piglets from the OEO sow group at T2 (0.227 ± 0.144 vs 0.108 ± 0.073). At the family level, the top most abundant families in piglet samples included *Ruminococcaceae* (0.185 ± 0.039), *Lachnospiraceae* (0.121 ± 0.065), *Enterobacteriacea*e (0.119 ± 0.083), *Lactobacillaceae* (0.104 ± 0.007), *Bacteroidacea*e (0.064 ± 0.083), *Prevotellaceae* (0.058 ± 0.051), *Peptostreptococcacea*e (0.047 ± 0.030), *Clostridiaceae* (0.043 ± 0.013), unclassified (0.041 ± 0.007) and *Erysipelotrichaceae* (0.032 ± 0.022), (Fig. 6d).

Taxonomic differences in piglets between timepoints irrespective of treatment group are shown in Additional File 6. At the genus level, most genera remained unclassified, however at the family level, *Lachnospiraceae* and *Bacteroidaceae* were significantly decreased in piglets between T1 and T2, while *Veillonellaceae, Erysipelotrichaceae* and *Prevotellaceae* were significantly increased at T2 compared to T1.

The effect of OEO on maternally supplemented piglets was analysed separately for each timepoint (LDA > 3.0). At T1, nine taxonomic families were more abundant in OEO piglets and eleven were less abundant compared to controls (Fig. 7b). Differences between control and piglets from OEO supplemented sows were more pronounced at T2 with fifteen families being increased and nineteen decreased (Fig. 7c). Despite many families remaining unclassified, the top five families which increased in piglets from OEO supplemented sows at T2 were: *Lachnospiraceae, Ruminococcaceae, Erysipelotrichaceae, Clostridiaceae* and *Spirochaetaceae.* The top 5 most decreased families in the OEO group compared to controls were*: Enterobacteriaceae, Enterococcaceae, Veillonellaceae, Barnesiellaceae* and *Tannerellaceae* (Fig. 7c).

## Discussion

The current study investigated the effects of OEO supplementation in sows on piglet performance and the faecal microbiota. The relationship between dam and offspring microbiome is complex and was beyond the aim and scope of the current study as we were unable to directly evaluate vertical microbial transfer. Therefore, the effects of OEO supplementation on sows and their piglets are discussed independently.

### Effect of OEO on sow performance

Supplementation of OEO to the sow as an unmixed top dress on the morning ration resulted in a trend for reduced voluntary feed intake on the first week of the study however, this did not persist past the first week of supplementation. As sows in this trial had no prior exposure to OEO or similar products, habituation may have been required. In contrast, previous work with the same OEO source [18] showed a trend for increased feed intake in the third week of lactation compared with un-supplemented control sows. In this case, the supplementation of sows throughout gestation and lactation may have helped to increase intakes during lactation due to olfactory or gustatory habituation prior to farrowing.

Late gestation and lactation are the most demanding periods for sows where energy and nutrient requirements significantly increase to support milk production and growth of their offspring. Despite increase in feed provision, nutrients from body reserves can often be used during lactation to meet this demand. Ji et al. [24] reported loss of backfat thickness between gestation and lactation associated with changes in plasma lipid and protein metabolism. In the current study, BCS as a measure of fat deposition in sows was recorded at the start of the study (T0) (~d110 of gestation), at farrowing and at the end of lactation. In a previous study, OEO supplementation showed no effect on sow backfat thickness between the end of gestation and weaning [18]. In contrast, the current study reports a decrease in BCS in control sows, while sows in the OEO supplemented group maintained a consistent BCS until the end of lactation, despite the same level of feed intake and exposure to environmental conditions. Discrepancies between these results may be explained by the difference in weaning age (26d vs 21d), whereby sows in this study were providing milk for heavier piglets over a longer period. Maintenance of body condition throughout gestation and lactation are key to support breeding performance in the following reproductive cycle, where fewer sows would be expected to return to oestrus (unsuccessful breeding event). In this trial, return to oestrus was similar for both treatment and control sows. However, as this trial was not designed or powered to look at this specific variable, a larger sample size would be required to provide sufficient replication to investigate the potential benefit of OEO on breeding and fertility performance which are important economic factors in pig production.

Recent studies have provided evidence to suggest that the gut microbiota is a major contributor to adiposity in pigs which has also been recognised in human and rodent models [25–27]. OEO may confer benefits for nutrient absorption or diet efficiency through modulation of the gut microbiota composition and/or function leading to a positive effect on energy balance, as reflected in the maintenance of BCS throughout lactation. Previous studies provide evidence to support a link between metabolism and microbiota [16, 18, 28], and bacterial taxa associated with OEO treatment such as *Lactobacillus, Fibrobacter* and *Akkermansia* support this.

### Effect of OEO on sow microbiota

*Lactobacillus* was the genus most increased in OEO supplemented sows. *Lactobacillus* are lactic acid producing bacteria that degrade fermentable carbohydrates into acetate and lactate which a) lower the pH of the gastrointestinal tract inhibiting the growth of potential pathogens, and b) provide substrates for cross-feeding and interaction with intestinal bacterial residents to produce butyrate [18, 29–31]. *Lactobacillus* spp. are amongst the most commonly used probiotic to improve growth performance, feed conversion efficiency, nutrient utilisation and gut health in pigs [32]. Previous studies have also reported increased *Lactobacillus* count in faeces of sows supplemented with OEO and this was associated with a reduction in *E. coli* and *Enterococcus* counts [18]. The current study also reports a decrease in *Enterobacteriaceae* in both sows and piglets from the OEO supplemented sow group and a decrease in *Enterococcus* in piglets from OEO supplemented sows. In other work, in growing-finishing pigs fed a reduced protein, amino acid supplemented diet, OEO showed increased *Lactobacillus* counts in the ileal digesta [28]. Finally, *Lactobacillus* counts were also increased in the caecum of broilers supplemented with a plant extract containing carvacrol, the main aromatic compound found in the source of OEO used in this study [33]. *Lactobacillus* and *Bifidobacteria* have both been recognised as important colonisers of the healthy infant microbiota [34], although OEO did not directly affect *Bifidobacteria* relative abundance, the effect of OEO on *Lactobacillus* population may be particularly beneficial considering that *Bifidobacteriaceae* were found to be severely reduced in all sows between pre-farrow and weaning.

Other bacterial families that were found to be increased in sows fed OEO are *Fibrobacteraceae* and *Akkermansiaceae*. *Fibrobacteraceae* is an important family of plant-based cellulose degrading bacteria that possesses glycoside hydrolase enzymes including xylanases [35, 36] that enable the breakdown of complex plant materials into fermentable oligosaccharides available for other species such as *Lactobacillus*. Members of *Akkermansiaceae* have demonstrated the ability to produce acetate and propionate as products of mucus degradation and have been suggested as biomarkers for a healthy intestine due to their role in gut barrier function, permeability and protection from intestinal inflammation [37–39]. Moreover, recent studies have detailed an inverse correlation between the abundance of *Akkermansiaceae* and several intestinal disorders, including inflammatory bowel disease, Crohn’s disease and ulcerative colitis in humans [36–38, 40]. *Finally, Akkermansia muciniphila* inversely correlates with obesity and diabetes in both mice and humans and was found to be high in the OEO supplemented sow group. Moreover, the mechanism of *A. muciniphilia* on weight loss and adipocyte reduction has been linked with anti-inflammatory properties [38, 41].

### Effect of OEO on piglet health and performance

Despite similar birth weight and litter size, piglets from OEO supplemented sows had significantly increased body weight and ADG in the first week of life. Piglets from OEO supplemented sows also showed reduced mortality and medication use compared to piglets from control sows. Piglet growth during the first few weeks of life is strongly dependent on milk quality and quantity however, these could not be recorded in the present study. While a previous study reported significant effect of OEO supplementation on milk content including a reduction in fat content, an increase in T-lymphocytes and higher energy intakes in piglets [18, 42], others found no significant effect of OEO on colostrum and milk production or composition [17]. In this study, maternal supplementation with OEO was found to have a positive impact on the lifetime performance of piglets. This may have been due changes in their early life microbiota from changes in maternal transfer. Supplementation of all piglets with OEO in the creep diet did not devalue the benefits seen but did not result in improved creep intakes as was hypothesised.

Cheng et al. [28] reported that OEO supplementation through the growing-finishing phase improved growth performance and nutrient digestibility by modulating intestinal bacteria, intestinal morphology, and anti-oxidative capacity of pigs. In the current study, OEO supplementation was evaluated when administered to the sows to influence the colonisation of bacterial community in early life piglets therefore, the long term impact may have diminished over time compared to a prolonged period of supplementation (through the growing phase for example). Overall lifetime performance was more difficult to measure due to the low retention of individual ear tags at later timepoints. Although results did not reach statistical significance, piglets from OEO supplemented sows showed numerically greater bodyweight at weaning, 10 weeks post-weaning and at slaughter which could help reduce the number of days to slaughter as weight gain was equivalent to ~ 3 days of production [43]. It was also noted that piglets from OEO supplemented sows showed less individual weight variation, according to dispersion, in terms of weight and number of days to slaughter. This is a desirable factor for more efficient livestock production.

The OEO used in this study has also been shown to increase growth efficiency in other livestock species, such as poultry, in challenged [20] and unchallenged conditions [44]. The current trial was run in commercial conditions and aimed to represent a practical setting in which the study result might be applied. Results from studies performed in such commercial environments may provide a better model for testing feed additives such as phytogenics and increase the transferability of the results to the industry compared to trials conducted in research facilities.

Piglets from OEO supplemented sows showed a significantly reduced number of medical interventions in response to health monitoring. Health records and interventions followed the standard operating procedures in line with welfare practices and veterinary recommendations for the farm. This suggests that the piglets from the OEO supplemented sows were in better health which may be linked to the improved growth performance seen in piglets during the nursing phase. This may be influenced by improved milk or colostrum provision, suckling behaviour, or energy conversion in the piglet. However, these links are only associative, and causality cannot be determined from the current study. The majority of health observations in this study were recorded as mild cases of ‘joint ill’: a sporadic and non-specific condition that affects young pigs and causes swollen joints due to opportunistic bacterial invasion from the bloodstream [45]. OEO has been shown to improve gut barrier function [16] and have antimicrobial properties [13, 46] however, the causative agent of this condition was not determined in the present study and further clinical investigation would be required to assess the impact on OEO on this disorder in pigs.

The pharmaceutical use of zinc in pig production is facing an agricultural ban within the EU for concerns over heavy metal contamination, AMR gene accumulation in the environment and associated risks to human health [47]. OEO has previously been suggested as a sustainable alternative for antimicrobials [48], in the current study zinc oxide was not used in weaning diets of any piglets. Therefore, the potential for OEO to replace zinc oxide in commercial weaning diets would warrant further investigation.

### Effect of OEO on the piglet microbiota

As with sows, microbiota analysis of piglet faeces used in this study revealed a high level of inter-individual diversity between piglets and timepoints. However, the piglet microbiota composition was clearly distinct from sows and included a much larger proportion of taxa that were unknown or uncharacterised at the family and genus level.

Taxonomic analysis also revealed that the piglet gut harbours a high relative abundance of *Proteobacteria; a* phylum that includes a wide variety of pathogenic bacteria. Previous studies have linked increases in *Proteobacteria* with a number of metabolic disorders and inflammatory gut conditions [49]. In this study, piglets from OEO supplemented sows had a reduced proportion of *Proteobacteria* compared to the control*.* This effect may reduce the disease risk due to a smaller pathogen reservoir. Although, piglets selected for microbiota sampling did not receive any direct medication at any point in the trial, exchange of microbiota with treated littermates was possible.

Analysis of the effect of maternal OEO supplementation on piglet microbiota revealed more taxa were affected at T2 than at T1. The relative abundance of families including *Spirochaetaceae, Peptostreptococcaceae* and *Lachnospiraceae* were increased in the piglets from OEO supplemented sows at both timepoints while *Ruminococcaceae* and *Erysipelotrichaceae* were only increased at T2 compared to controls. In control piglets, *Rikenellaceae, Marinifilaceae* were increased at T1 while *Enterobacteriaceae, Veillonellaceae* and *Barnesiellaceae* were increased at T2 compared to piglets from OEO supplemented sows. The biological relevance of some of these families on the host remains unclear or unknown, others have been found to be associated with health and disease resilience, energy utilisation, growth performance and inflammation management. For example *Ruminococcaceae* and *Lachnospiraceae* are both involved in the digestion of dietary polysaccharides (e.g. fibre, cellulose and lignin) resulting in the production of SCFA including butyrate [50, 51]. Butyrate represents a major energy source for intestinal epithelial cells and is known to enhance barrier function and attenuate intestinal inflammation [52]. Quan et al. [53] found that pigs with high feed efficiency had enriched OTUs from the *Ruminococcaceae* family compared to pigs with lower feed efficiency. The higher abundance of these families in piglets from OEO supplemented sows may have contributed to the increased performance seen in this trial. In addition, previous studies have shown that diarrheic piglets had lower abundance of *Lachnospiraceae* and *Ruminococcaceae* compared to healthy piglets [54] and patients suffering from IBD and acute colitis consistency show depleted *Lachnospiraceae* and *Ruminococcus* spp. [55] suggesting that these organisms are important in maintaining intestinal homeostasis.

Interestingly, as we see an increase in butyrate-producer families in piglets from OEO supplemented sows, we also see a decrease in *Enterobacteriaceae* compared to control piglets. It has been shown that the absence of a healthy butyrate-producing microbiota leads to an increased nitric oxide synthase (NOS2) gene expression and nitrate production which favours the bloom of *Enterobacteriaceae* [56]*.* Relative abundance of *Enterobacteriaceae has been positively correlated with a large number of intestinal inflammatory disorders and also linked to stress.* In piglets, weaning is a critical period of production which has been associated with activation of the gut-brain axis leading to intestinal inflammation and increased gut permeability [57]. These changes can lead to opportunistic proliferation of *Enterobacteriaceae*, diarrhoea, increased requirement for antibiotic treatment, poor performance and economic losses. Therefore, by reducing *Enterobacteriaceae* abundance and promoting a higher butyrate-producing bacterial community before weaning, OEO may beneficially affect piglets during the weaning transition stage.

Another notable family of interest was *Veillonellaceae*, which was increased in piglets from the control group. Members of the *Veillonellaceae* family can act as opportunistic pathogens and be responsible for polymicrobial infections with several isolates reported to carry multiple resistance to antimicrobial agents [58]. As piglets from the control group displayed significantly more signs of poor health including swollen joins that are caused by opportunistic bacterial invasion, the increase in *Veillonellaceae* in the control group may have been linked with this and therefore warrants further investigation.

Additionally, *Peptostreptococcaceae* and *Spirochaetaceae* were amongst the families increased in piglets from OEO supplemented sows. Although these families are normal components of the pig gut microbiota [6, 59], their presence may be associated with undesirable effects due to the pathogenicity of specific members of their group [60]. However, health and performance results showed that piglets from OEO supplemented sows were not negatively impacted by their presence and in fact showed better health than controls*. Erysipelotrichaceae* was also amongst the families increased in piglets from the OEO supplemented sow group. Members of the *Erysipelotrichaceae* family have been associated with lower feed efficiency in pigs [61], but higher feed efficiency in calves [62]. Therefore, specific genus and species difference may play an important role in these cases and highlight the limitation of the 16S rRNA amplicon sequencing approach. To further our understanding of the effect of OEO in piglet and sow microbial communities, a metagenomic approach or full 16S rRNA gene sequencing would enable the characterisation of taxonomy at a deeper level and more importantly to determine the effect of OEO on the microbiome at a functional level. A metabolomics approach would also represent an interesting route for further investigation especially regarding the production of butyrate and other SCFA.

In this study sow supplementation with OEO has been shown to have positive effects on piglet health and performance in a commercial environment which extended past the weaning period. By targeting the microbiome, OEO has shown scope to be considered a sustainable tool for the livestock industry to reduce the reliance on antimicrobials without compromising animal performance or welfare. Timing of intervention strategy is key. This study noted that more than 75% of piglet mortalities occurred before 5 days of age, therefore targeting early gut development via maternal supplementation may represent a strategic window of opportunity to support this vulnerable life stage. Although vertical transfer of microbiota from sow to piglet is likely to play a strong role in the results observed, further mechanistic studies would need to be performed to ascertain specific maternal factors responsible for the cause and effects observed in piglet performance (milk, microbiota transfer, etc.). Increased understanding of the mode of action of feed additives such as OEO will improve and refine their application in the future.

## Conclusions

This trial concludes that the inclusion of OEO supplementation to maternal rations during late gestation and lactation can lead to improvements in progeny health and performance with a reduced incidence of mortality and lower need for medication. Early piglet growth was significantly improved pre-weaning, and numerically improved post-weaning with less variation in bodyweights seen at each timepoint. This suggests that maternal supplementation can affect lifetime piglet performance. Maternal OEO supplementation did not increase piglet intake of creep containing OEO before weaning. Thereby plant based phytobiotics can provide livestock producers with a nutritional tool for the improvement of sow and progeny health and performance, creating beneficial changes in the microbiota with significant effects on lifetime performance and reduced medication use. This could help to improve animal welfare while reducing the reliance on antimicrobials and support animal production profitability.

## Methods

**Fig. 8:**
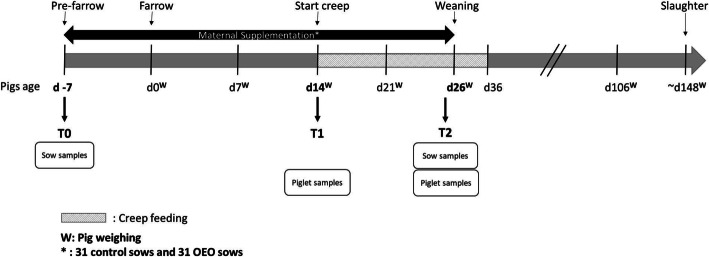
Experimental design

In a blinded study, sixty-two multiparous sows across two farrowing batches were randomly allocated to control or OEO supplementation. Treatment was top dressed to the sow diet daily from seven days prior to farrowing until weaning (~ 26 days). At two weeks of age, piglets from all treatment groups were offered creep containing OEO. Faecal samples were collected from sows and piglets and analysed for 16S rRNA gene sequencing, see Fig. 8 for experimental design. The aim of this study was to understand the effects of OEO supplementation on sow and piglet microbiota through maternal supplementation and associated effects on performance, health, and efficiency.

### Animals, diet, and experimental conditions

The blinded trial was carried out on a farrow-to-finish indoor commercial pig unit in Lincolnshire (UK). The performance of this unit was in line with the top 10% of UK industry before the start of the trial [63]. Sixty-two Landrace x Large White sows and their piglets (869) were followed over two consecutive batches between July and August 2018, in a weekly batched farrowing system. All routine farm procedures were followed as per existing practice such as vaccination schedule, piglet processing for iron injection, teeth clipping and tail docking. However, other gut health products were removed for the duration of the trial and no antibiotics or therapeutics were included in either sow or piglet diets (e.g. zinc oxide). All feeds were manufactured and supplied by Porters Animal Feeds (Navenby, UK). Sows were group-housed during gestation and moved to individual farrowing pens approximately one week prior to expected farrowing date (day 109–111 of gestation), where they were balanced for parity and BCS and randomly allocated to either the control or OEO (Orego-Stim®, Anpario ltd. Worksop, UK) treatment. Experimental design can be seen in Fig. 8.

Farrowing pens were concrete solid floor with straw and had a piglet creep box to the front of the sow, providing an area with a heat pad, feeder, and water. Environmental conditions were recorded at three locations in each farrowing house manually twice a day and via a data logger (RS, Lascar EL-USB-2) every 4 h (Additional File 3).

Upon entering the farrowing accommodation, sows were fed a lactation diet (CP: 18.2% and ME: 17.3 MJ/kg) manually twice daily, according to the Stotfold scale (a commercial feeding strategy for sows). Sows in the treatment group were supplemented with OEO at a daily rate 15 g top dressed in the morning ration (1% OEO (*Origanum vulgare ssp. hirtum)* on an inert carrier). The control group followed the same conditions but were supplemented with carrier only (15 g inert calcium carbonate carrier). Supplementation was administered daily throughout the whole lactation period up to weaning (26 days post-farrowing). All sows and piglets had ad lib access to water.

Daily feed intake and refusals were recorded for each sow throughout lactation. Body condition score was assessed at entry into the farrowing house, at farrowing and at weaning according to the criteria developed by Patience and Thacker [21]. At farrowing, number of piglets (total, born alive, born dead, mummified) and assisted farrowing interventions were recorded.

All viable piglets were ear tagged within 24 h of birth with a unique identification number and colour coded according to maternal treatment. All piglets were individually weighed weekly from birth to weaning (Brecknell Digital Handheld Scale with an accuracy of +/− 0.01 kg from birth to 2 weeks, Pharmweigh trolley scale with accuracy of +/− 0.1 kg from 3 weeks to weaning). If required, cross-fostering only occurred between litters from the same treatment group. Cross fostered or medicated piglets were excluded from microbiota sampling. Health and medication use per animal were recorded throughout the trial and administered as per standard farm practice. Piglet pre-weaning mortality was recorded between birth and weaning. Faecal scores were recorded per pen, weekly, between week 2 and weaning (See Additional File 4 for faecal scoring scale).

At 14 days of age, creep feed supplemented with OEO was offered daily to piglets from all sows (control and OEO treatment). Supplementation level in creep feed was 1 kg/t of OEO (5% OEO (*Origanum vulgare ssp. hirtum)*). Creep feed (CP: 20.7% and ME: 17.4 MJ/kg) was made in a single 3 t batch to ensure homogeneity and no zinc oxide was included. Creep feed intake per litter was measured weekly and estimated per piglet per day.

At weaning (26 days), sows were removed, and piglets moved to a mixed treatment weaning pen (60–72 pigs per pen) according to sex and size. Weaned pigs received the same creep diet (containing 1 kg/t OEO) for the first 10 days post-weaning followed by standard commercial diets with no OEO supplementation. Pigs were weighed again at 10 weeks post-weaning and at slaughter.

### Sample collection for microbiota analysis

Fresh faecal samples were collected from sows at the entry into farrowing house (T0) and on the day prior to weaning (25 days post-farrowing) (T2). Fresh faecal samples were collected from rectal swabs of two piglets per litter at 14 days of age (T1) and on the day prior to weaning (T2). Faecal swabs were immediately preserved in DNA shield (Zymo, Research, Irvine, US). Samples were stored at − 80 °C until DNA extraction and amplicon sequencing. Choudhury et al. [64], have shown that rectal swabs and faecal samples provide comparable insight for the microbiota of young piglets and provide a useful tool due to the challenging nature of obtaining fresh faecal samples from suckling piglets [64]. Piglets selected for sampling were randomly chosen from those with bodyweights close to the mean litter weight. The same piglets were followed over time and excluded any cross-fostered or medicated piglets.

### 16S rRNA gene amplification and sequencing

The experimental protocol followed the general recommendations set out in Pollock et al. [65]. Faecal DNA from sows (at T0 and T2) and piglets (at T1 and T2) was extracted using the PowerSoil kit (Qiagen, Hilden, Germany) in accordance to the manufacturer’s instruction including an initial bead beating step of 3x40s at 6,500 rpm (Precelly homogenizer, Bertin, France). A blank control (0.2 g of water instead of faeces) was also extracted alongside the samples and followed the same procedure. A mock community mix of 20 genomic strains was also included (ATCC® MSA-1002™, Manassas, VA, USA). DNA was quantified using the Qubit 4 Fluorometer (Thermo Fisher Scientific, Waltham, MA, USA) and normalised to 5 ng/μl.

16S rRNA libraries were prepared from amplifying the V3-V4 16S rRNA hypervariable region using the 341F and 534R primers and adding sequencing adapters and dual-index barcodes to the amplicon [66]. Firstly, the V3-V4 hypervariable region of the 16S rRNA gene was amplified using the Kapa Hifi HotStart Readymix (Kapa Biosystems; Boston, MA) with forward primer 5′-TCGTCGGCAGCGTCAGATGTGTATAAGAGACAGCCTACGGGNGGCWGCAG-3′ and reverse primer 5′-GTCTCGTGGGCTCGGAGATGTGTATAAGAGACAGGACTACHVGGGTATCTAATCC-3′. Amplification cycling parameters consisted of 95 °C for 3 min, followed by 25 cycles of 95 °C for 30 s, 55 °C for 30 s, 72 °C for 30 s, and a final elongation step of 72 °C for 5 min. PCR amplicons were cleaned up using AMPure XP beads (Beckman-Coulter; Fullerton, CA) following the manufacturer’s instructions, and visualized on an Agilent 4200 TapeStation system (Agilent Technologies; Palo Alto, CA) to confirm amplicon size. Secondly, index PCR was performed to attach dual indices and sequencing adapters to the amplicons using the Nextera XT index kit (Illumina, San Diego, CA) using the following program: 95 °C for 3 min, followed by 8 cycles of 95 °C for 30 s, 55 °C for 30 s, 72 °C for 30 s, and a final elongation step of 72 °C for 5 min. PCR products were again cleaned up using AMPure XP beads and visualized on the Agilent TapeStation system before final library quantification (Qubit dsDNA High Sensitivity Kit, Thermo Fisher; USA).

Finally, all samples were pooled into equimolar concentrations and sequenced using paired-end sequencing (2 × 300 bp) on the Illumina MiSeq platform (Illumina; San Diego, CA). PhiX control spike was added at 10%. All raw sequence reads are available in the NCBI SRA database under project accession number PRJNA637866.

### 16S rRNA gene sequencing quality control and processing

Sequencing output resulted in 34,426,016 raw reads. Raw reads were trimmed for Illumina Nextera XT adapters and read-through using Trimmomatic version 0.38 [67]. Adapter trimmed reads were checked for quality through FastQC version 0.11.7 (https://github.com/s-andrews/FastQC) for adapter sequences and average sequencing quality drop off below Phred 20. Adapter trimmed reads were hard trimmed in the 3′ to 5′ direction for each forward and reverse read by 16 and 60 bp respectively, removing poor quality read ends. Reads were further curated through Sickle version 1.33 [68] trimming reads with base quality below Phred 20 and removal of reads less than 236 bp in length. Read curation resulted in a total of 16,189,792 high quality reads.

Demultiplexed curated sequencing reads were imported into the QIIME2 platform version 2018.11 [69]. Samples were denoised into amplicon sequence variants (ASVs) through DADA2 [70] which simultaneously corrects sequencing reads, filters out PhiX contaminant reads, filters chimeric sequences, filters singleton reads, merges paired-end reads, and dereplicates resulting sequences.

To classify the ASVs, a custom trained naïve Bayesian classifier was created using the *q2-feature-classifier* trained to the V3-V4 region of the 16S rRNA gene from the 16S SILVA database (release 132) using the 99% identity representative sequences. This was done due to the improved accuracy of taxonomic classification by this methodology [71]. Briefly, the 99% representative sequences were imported into QIIME2 and the *qiime feature-classifier extract-reads* plugin was used to extract the V3-V4 reference reads using the V3-V4 target primers (forward primer CCTACGGGNGGCWGCAG; reverse primer GACTACHVGGGTATCTAATCC). The naïve Bayesian classifier was trained using the extracted reference V3-V4 reads through the *feature-classifier fit-classifier-naive-bayes* plugin.

A BIOM-format table containing the samples, classified ASVs, and frequencies was exported for data visualisation in microbiome analyst [72–74].

### Statistical analysis

#### Health and performance data

Power calculation determined the sow sample size at *n* = 31 to detect a 10% difference in performance based on 80% statistical power and α value of 0.05. Statistical analyses were carried out using IBM SPSS Statistics (v24) using a significance level of *p* ≤ 0.05 and *p* ≤ 0.1 for statistical trend. Four litters were excluded from the trial (1 from OEO and 3 from control group) due to illness in one sow and mixed fostering. Analysis of the effect of OEO on performance data was carried out using a linear mixed model (with treatment as a fixed factor) but also included sow, batch, and sex as random factors where appropriate. Values reported indicate means ± standard error (SE) unless otherwise stated. Value represented with differing letters indicate statistically significant differences between groups.

#### Microbiota data

A phylogenetic tree was constructed using the *align-to-tree-mafft-fasttree* QIIME2 pipeline from the *q2-phylogeny* plugin. Briefly, all sample representative sequences were aligned by MAFFT [75] where ambiguously aligned regions were masked to improve phylogenetic inference. A phylogenetic tree was constructed using FastTree 2 [76] and rooted to the midpoint for the purpose of alpha diversity calculations (Pielou’s Evenness, Faith’s Phylogenetic Diversity and Shannon’s diversity index).

Samples were rarefied at an equal depth to reduce bias to a sample depth of 7368 as this was the lowest sample depth above the blank control [77]. The *core-metrics-phylogenetic* pipeline was run through the QIIME2 *q2-diversity* plugin computing alpha diversity statistical analysis [69]. Bray-Curtis dissimilarity and Unweighted UniFrac distance was calculated using the *core-metrics-phylogenetic* pipeline through the QIIME2 q2-diversity plugin and rarefied to a sample depth of 7368 as previously described [78].

Statistical analysis of alpha diversity indices was performed using Kruskal-Wallis rank-sum test and beta diversity using PERMANOVA (Permutational multivariate analysis of variance) analysis. Linear discriminant analysis (LDA) effect size (LEfSe) analysis was used to identify the differential family and genus between timepoint and between OEO and control groups. Microbiome analyst was used for data visualisations [72, 73], using low count filter set at a minimum of 4, prevalence in sample of 10% and low variance filter at 5% based on interquartile range.

## Supplementary Information

**Additional file 1.** Baseline trial data for all sow farrowing events.

**Additional file 2.** Body condition data for all sows and the scale used, as adapted from Patience and Thacker [21].

**Additional file 3.** Temperature log from data loggers in farrowing rooms as well as the weather as recorded for the region by BBC weather.

**Additional file 4.** Piglet weekly faecal scores (median values) and scoring scale.

**Additional file 5.** Principal coordinates analysis (PCoA) plots based on Bray-Curtis distances collated by sampling timepoint and treatment for a) sows and b) piglets.

**Additional file 6.** Linear discriminant analysis effect size (LEfSe), compiled by sampling timepoints at family and genus level for a) sows and b) piglets. Only taxa with absolute LDA score of > 3.0 and q-value < 0.1 (FDR) are displayed.

## Data Availability

All data used or analysed during this study are included in this published article and its supplementary information files. All raw sequence reads are available in the NCBI SRA Project number PRJNA637866 https://www.ncbi.nlm.nih.gov/bioproject/637866

## Abbreviations

16S rRNA: 16 Svedberg ribosomal Ribonucleic Acid
ADG: Average Daily Gain
AMR: Antimicrobial Resistance
ASVs: Amplicon Sequence Variants
BCS: Body Condition Score
CP: Crude Protein
LDA: Linear Discriminant Analysis
LEfSe: Linear discriminant analysis effect size
ME: Metabolisable Energy
NOS2: Nitric Oxide Synthase
OEO: Oregano Essential Oil
OTU: Operational Taxonomic Unit
PCoA: Principal Coordinates Analysis
PCR: Polymerase Chain Reaction
PERMANOVA: Permutational Multivariate Analysis of Variance
UniFrac: Unique fraction
SE: Standard Error
SCFA: Short Chain Fatty Acid
t: Tonne
