# Supplementary material for: Oregano essential oil improves piglet health and performance through maternal feeding and is associated with changes in the gut microbiota
Source: Anim Microbiome. 2021 Jan 4;3:2. doi: 10.1186/s42523-020-00064-2 (PMC7934403; doi:10.1186/s42523-020-00064-2)
Supplement: Supplementary file 1 — Additional file 1. Baseline trial data for all sow farrowing events. [file 42523_2020_64_MOESM1_ESM.docx]

# Additional File 1

**Baseline trial data**

|  | Control | | Treatment (OEO) | |
| --- | --- | --- | --- | --- |
|  | Mean (%) | SE | Mean (%) | SE |
| N total served | 31 |  | 31 |  |
| N after exclusion | 28 |  | 30 |  |
| Parity | 4.03 | 0.408 | 3.42 | 0.398 |
| % CV birth weight | 19.41 | 1.28 | 20.35 | 0.97 |
| Born alive | 14.68 | 0.625 | 14.42 | 0.490 |
| Born dead | 1.00 | 0.185 | 0.87 | 0.257 |
| Mummified | 0.32 | 0.126 | 0.32 | 0.097 |
| Assisted farrowing | 8/31^a^ (25.8%) |  | 4/31^b^ (12.9%) |  |
| Oxytocin use | 4/31 (12.9%) |  | 1/31 (3.2%) |  |
| Dead <24h | 0.61 | 0.15 | 0.58 | 0.25 |
| Total number piglet tagged birth | 441 |  | 428 |  |
| Male/female | 239/202 |  | 217/211 |  |
| Number piglet cross fostered | 144 |  | 126 |  |
| Number weaned | 12.14 | 0.32 | 12.17 | 0.22 |
| Age at weaning (day) | 25.96 | 0.34 | 26.13 | 0.41 |

*OEO; Oregano Essential Oil*

*SE; Standard error*

*Mummified; piglets which are not fully formed in-utero*

*Assisted farrowing; Intervention was provided where necessary according to the farm standard welfare procedures*

*Oxytocin; administered according to the farm standard welfare procedures*
