# Supplementary material for: Oregano essential oil improves piglet health and performance through maternal feeding and is associated with changes in the gut microbiota
Source: Anim Microbiome. 2021 Jan 4;3:2. doi: 10.1186/s42523-020-00064-2 (PMC7934403; doi:10.1186/s42523-020-00064-2)
Supplement: Supplementary file 2 — Additional file 2. Body condition data for all sows and the scale used, as adapted from Patience and Thacker [21]. [file 42523_2020_64_MOESM2_ESM.docx]

# Additional File 2

**Sow Body Condition score**

Sow body condition scores (BCS) and scale used, scoring was adapted from Patience and Thacker (1989).

*OEO; Oregano Essential Oil*


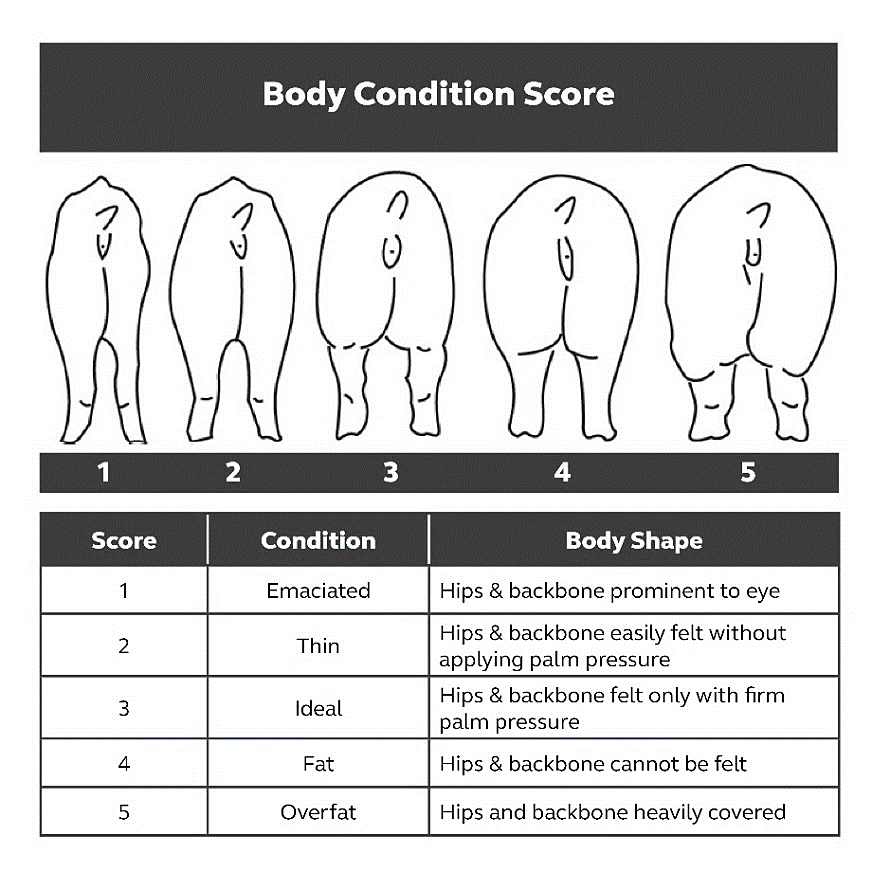


| P2 Back-fat* (mm) | Score | Condition | Body Shape |
| --- | --- | --- | --- |
| <15 | 1 | Emaciated | Hips and backbone prominent to eye |
| 15-18 | 2 | Thin | Hips and backbone easily felt without applying palm pressure |
| 18-20 | 3 | Ideal | Hips and backbone felt only with firm palm pressure |
| 20-23 | 4 | Fat | Hips and backbone cannot be felt |
| >23 | 5 | Overfat | Hips and backbone heavily covered |
