# Supplementary material for: Oregano essential oil improves piglet health and performance through maternal feeding and is associated with changes in the gut microbiota
Source: Anim Microbiome. 2021 Jan 4;3:2. doi: 10.1186/s42523-020-00064-2 (PMC7934403; doi:10.1186/s42523-020-00064-2)
Supplement: Supplementary file 3 — Additional file 3. Temperature log from data loggers in farrowing rooms as well as the weather as recorded for the region by BBC weather. [file 42523_2020_64_MOESM3_ESM.docx]

# Additional File 3

**Temperature log from each of the farrowing rooms**

|  | **Left** | | **Middle** | | **Right** | |
| --- | --- | --- | --- | --- | --- | --- |
| **Room 2** | Temp | Humidity | Temp | Humidity | Temp | Humidity |
| Average | 21.58 | 60.95 | 21.45 | 60.79 | 21.69 | 61.05 |
| Maximum | 31.5 | 82.5 | 29.0 | 80.0 | 29.5 | 78.5 |
| Minimum | 12.5 | 43.0 | 14.5 | 44.0 | 15.0 | 42.5 |
|  | **Left** | | **Middle** | | **Right** | |
| **Room 4** | Temp | Humidity | Temp | Humidity | Temp | Humidity |
| Average | 21.91 | 61.28 | 21.67 | 66.91 | 22.08 | 62.96 |
| Maximum | 29.0 | 83.0 | 29.0 | 86.5 | 30.0 | 82.0 |
| Minimum | 16.5 | 42.0 | 17.0 | 43.5 | 17.5 | 39.5 |

**Weather temperature range - BBC weather**

[www.bbc.co.uk/weather](http://www.bbc.co.uk/weather) accessed 1/09/2018
