# Supplementary material for: Oregano essential oil improves piglet health and performance through maternal feeding and is associated with changes in the gut microbiota
Source: Anim Microbiome. 2021 Jan 4;3:2. doi: 10.1186/s42523-020-00064-2 (PMC7934403; doi:10.1186/s42523-020-00064-2)
Supplement: Supplementary file 4 — Additional file 4. Piglet weekly faecal scores (median values) and scoring scale. [file 42523_2020_64_MOESM4_ESM.docx]

# Additional File 4

**
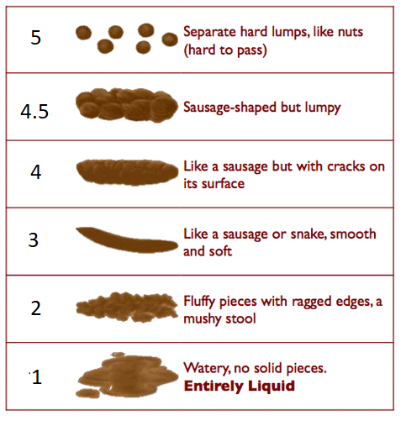
Piglet weekly faecal scores (median values) and scoring scale**

|  | Control | Treatment (OEO) | Number of piglets measured |
| --- | --- | --- | --- |
| Faecal scores piglets week 2 | 4.0 | 4.0 | 28 |
| Faecal scores piglets week 3 | 3.5 | 4.0 | 53 |
| Faecal scores piglets week 4 | 3.0 | 4.0 | 58 |

*OEO; Oregano Essential Oil*

**Piglet weekly faecal scores**


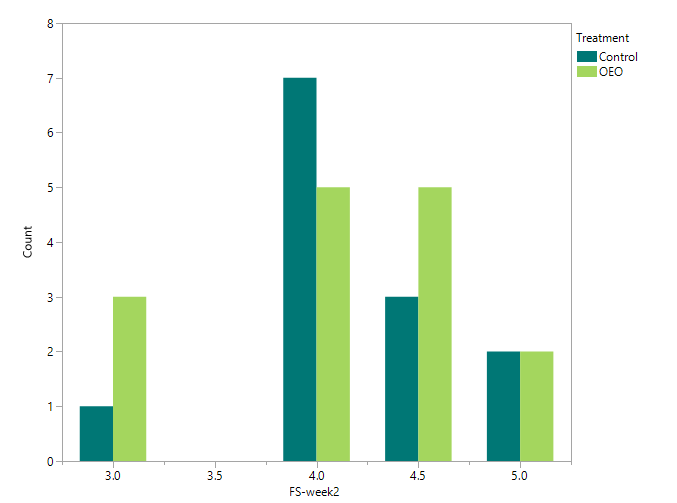

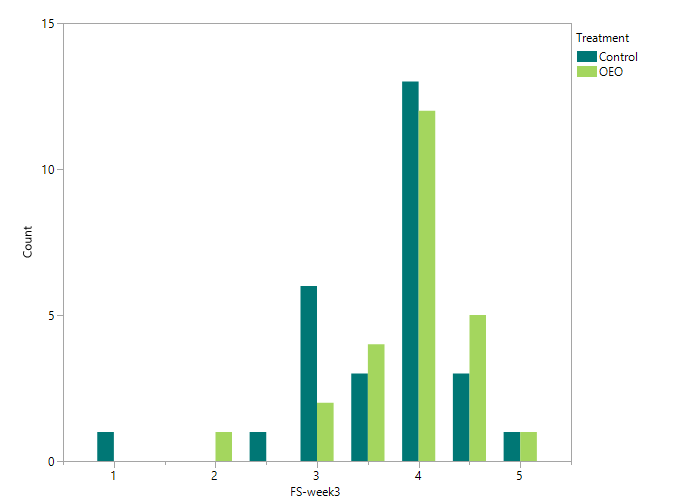

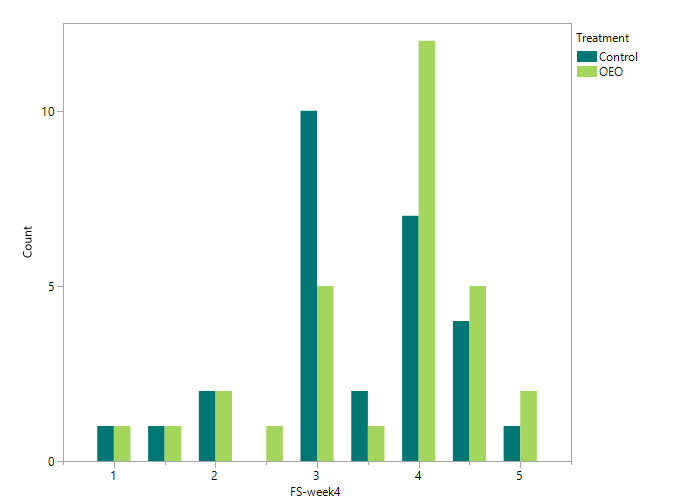


*OEO; Oregano Essential Oil*
