# Supplementary material for: Oregano essential oil improves piglet health and performance through maternal feeding and is associated with changes in the gut microbiota
Source: Anim Microbiome. 2021 Jan 4;3:2. doi: 10.1186/s42523-020-00064-2 (PMC7934403; doi:10.1186/s42523-020-00064-2)
Supplement: Supplementary file 5 — Additional file 5. Principal coordinates analysis (PCoA) plots based on Bray-Curtis distances collated by sampling timepoint and treatment for a) sows and b) piglets. [file 42523_2020_64_MOESM5_ESM.docx]

# Additional file 5


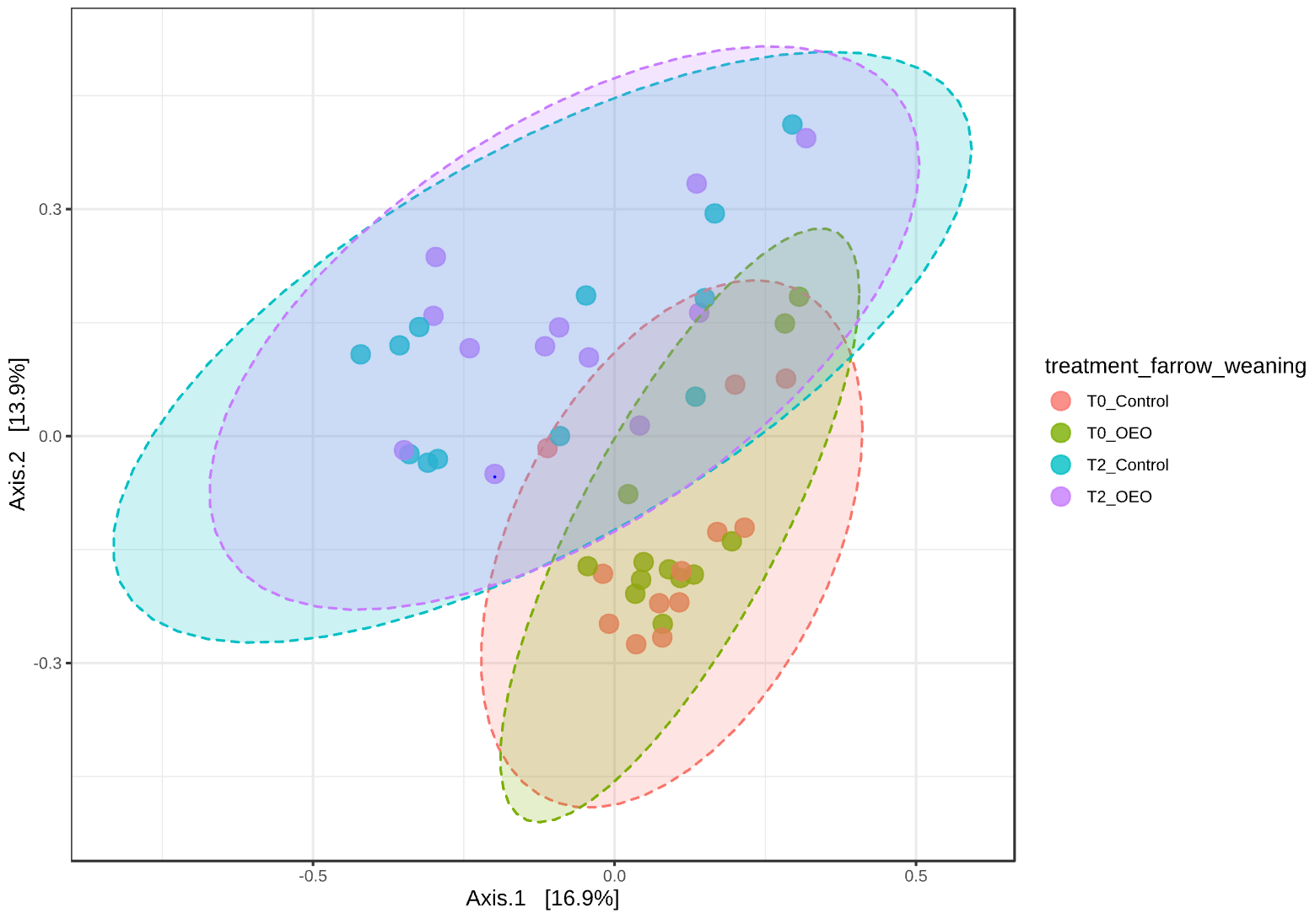
**Principal coordinates analysis (PCoA)** **plots based on Bray-Curtis distances**

Collated by sampling time point and treatment for a) sows and b) piglets.

1. Sows

*T0: Pre-farrowing, T2: 25 days post-farrowing*


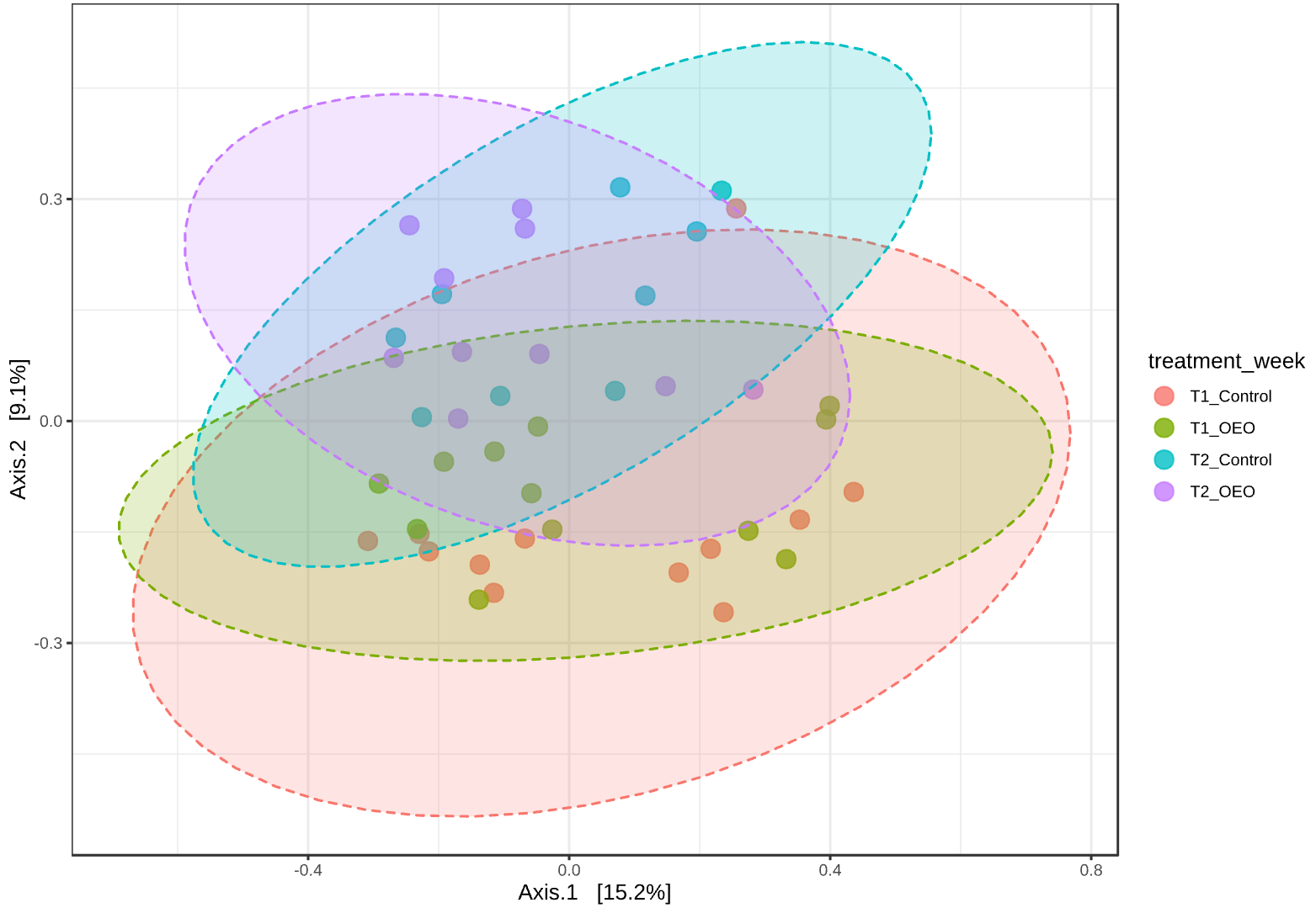
*OEO; Oregano Essential Oil*

1. Piglets

*T1: 14 days post-farrowing, T2: 25 days post-farrowing*

*OEO; Oregano Essential Oil*
