# Supplementary material for: Oregano essential oil improves piglet health and performance through maternal feeding and is associated with changes in the gut microbiota
Source: Anim Microbiome. 2021 Jan 4;3:2. doi: 10.1186/s42523-020-00064-2 (PMC7934403; doi:10.1186/s42523-020-00064-2)
Supplement: Supplementary file 6 — Additional file 6. Linear discriminant analysis effect size (LEfSe), compiled by sampling timepoints at family and genus level for a) sows and b) piglets. Only taxa with absolute LDA score of > 3.0 and q-value < 0.1 (FDR) are displayed. [file 42523_2020_64_MOESM6_ESM.docx]

# Additional file 6

**Linear discriminant analysis effect size (LEfSe)**

Compiled by sampling time points at family and genus level for a) sows and b) piglets. Only taxa with absolute LDA score of >3.0 and q-value <0.1 (FDR) are displayed.


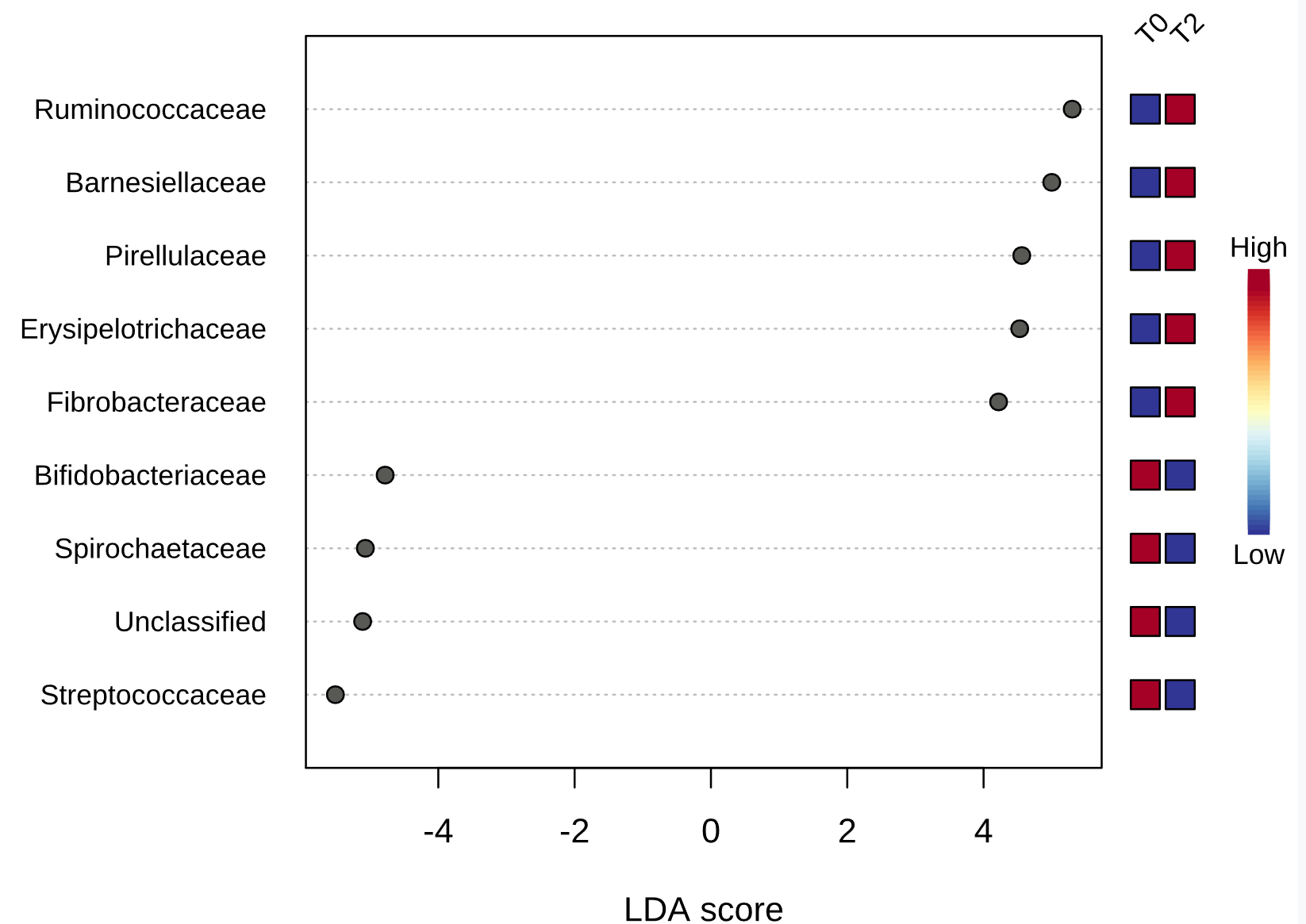

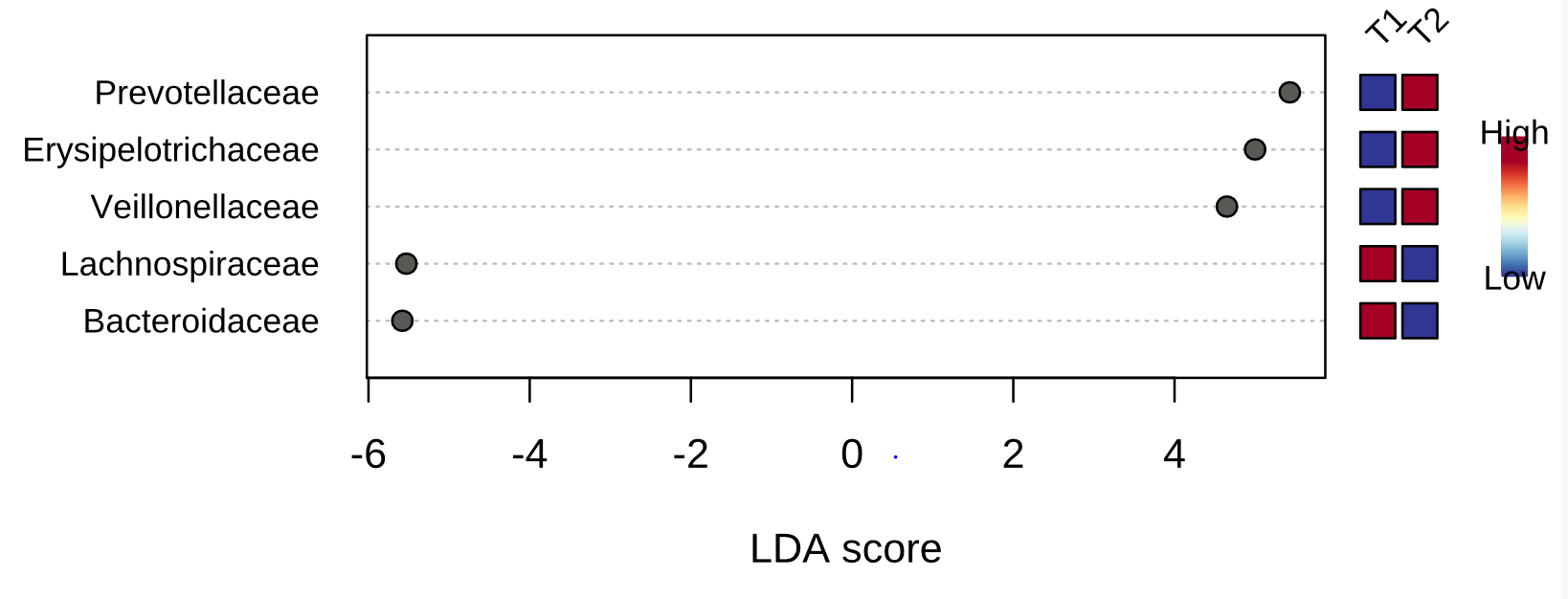


1. *b)*

**LEfSe Family level**


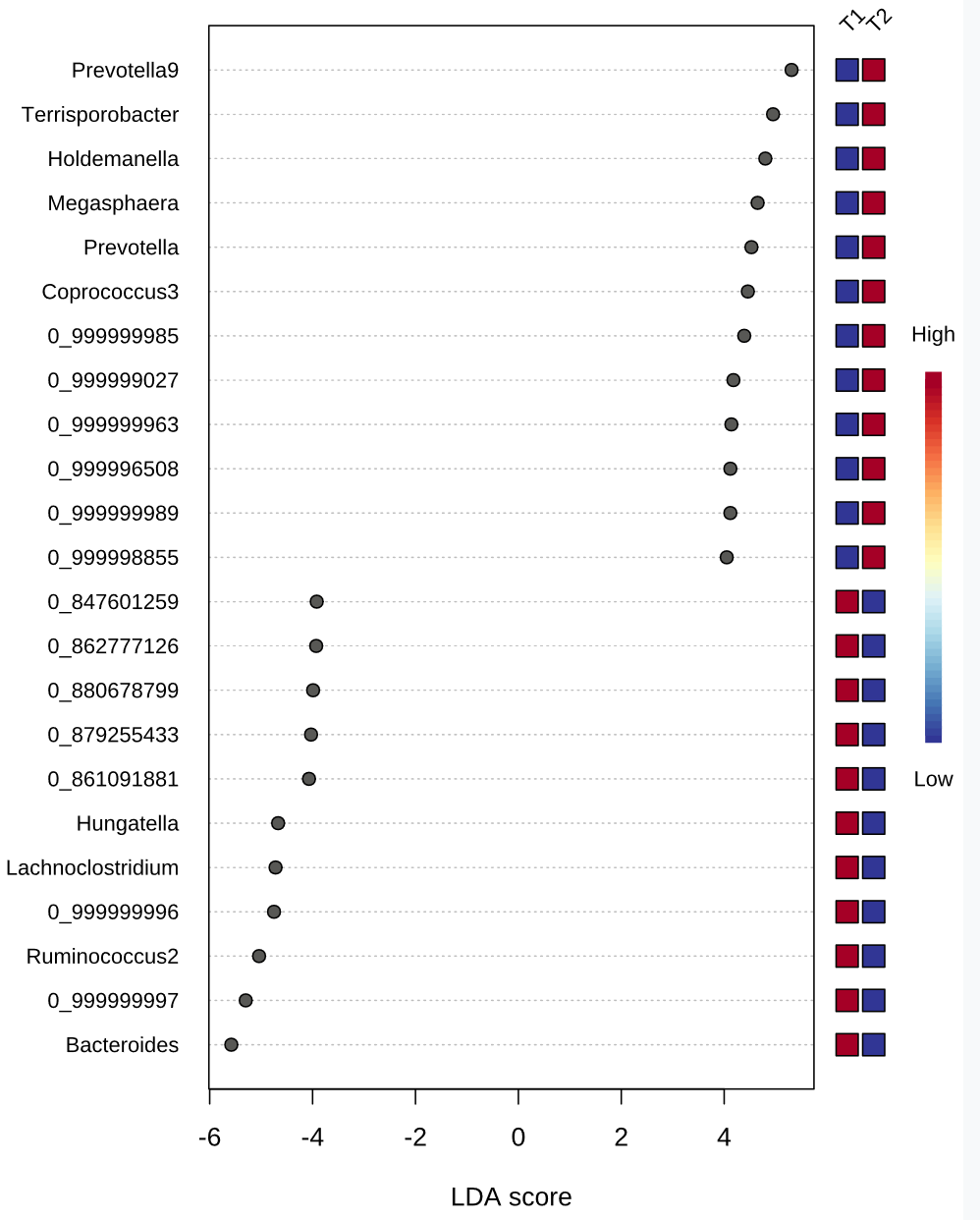
*T0: Pre-farrowing, T1: 14 days post-farrowing, T2: 25 days post-farrowing.*


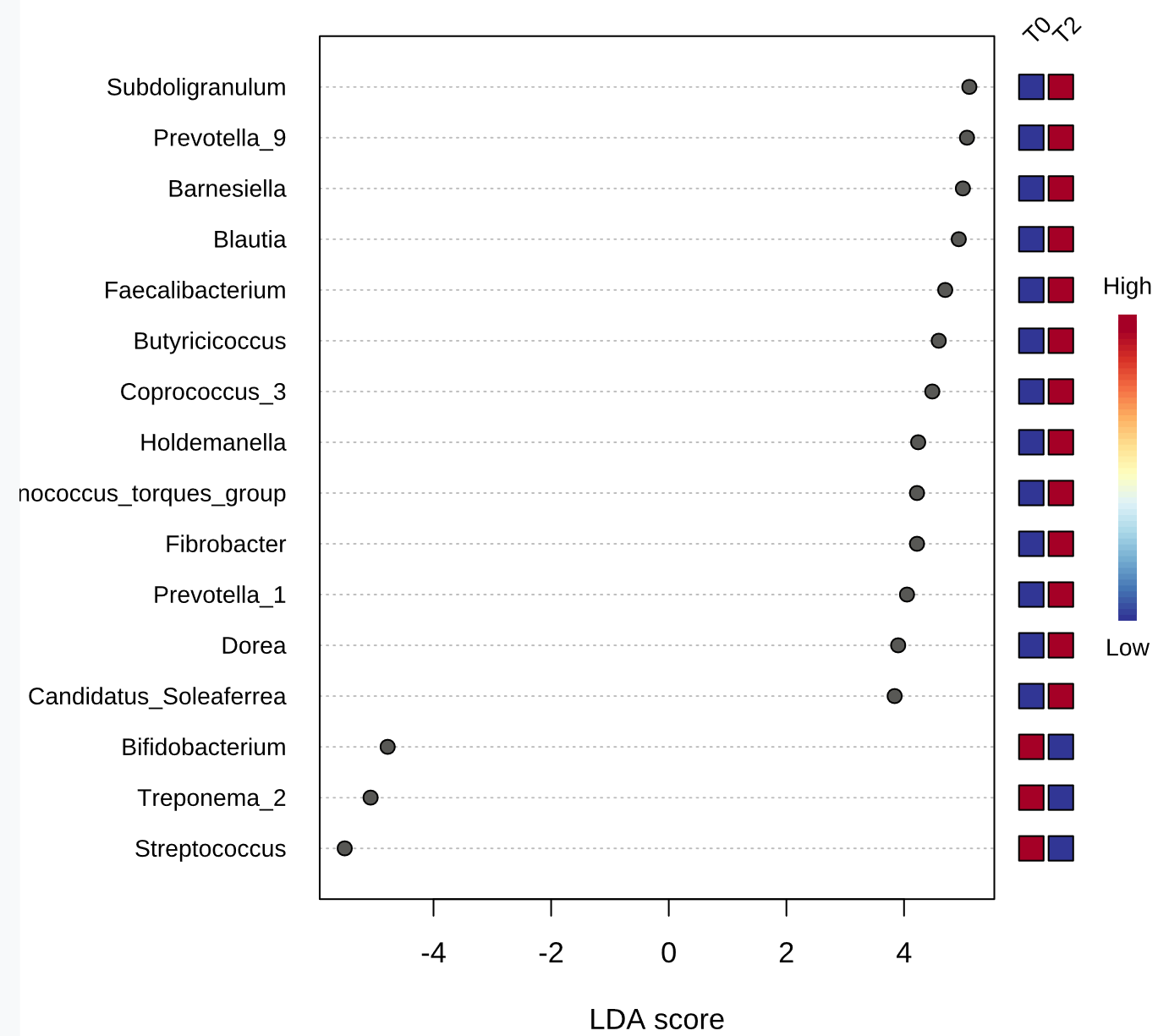


1. *b)*

**LEfSe Genus level**

*T0: Pre-farrowing, T1: 14 days post-farrowing, T2: 25 days post-farrowing.*
